# Supplementary material for: Comparative Transcriptome Analysis Identifies Key Defense Genes and Mechanisms in Mulberry (Morus alba) Leaves against Silkworms (Bombyx mori)
Source: Int J Mol Sci. 2022 Nov 4;23(21):13519. doi: 10.3390/ijms232113519 (PMC9656888; doi:10.3390/ijms232113519)
Supplement: Supplementary file 1 [file ijms-23-13519-s001.zip › Supplementary Figures.pdf]

Supplementary Figures

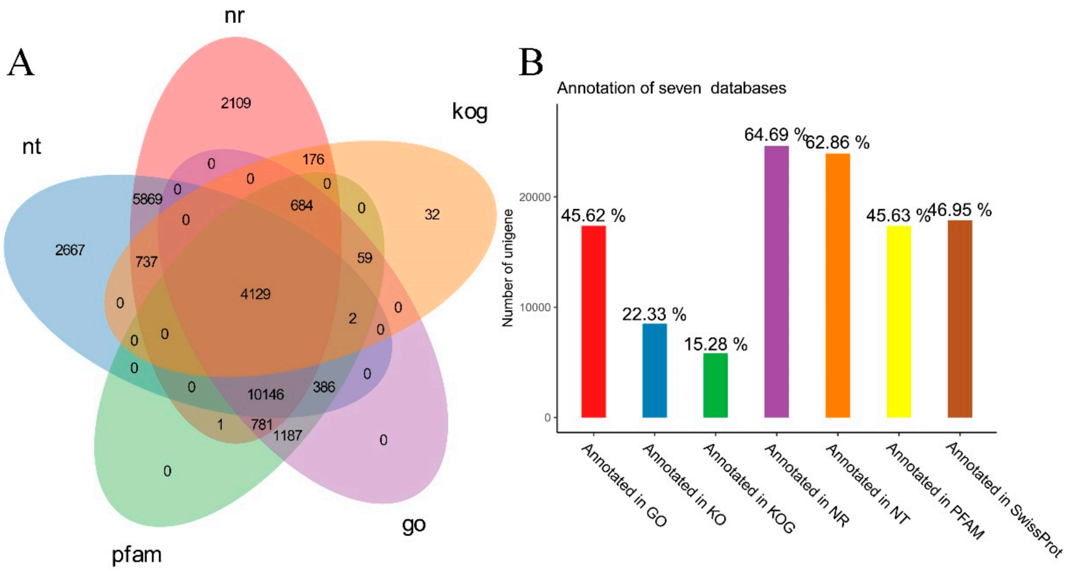

Figure S1. (A) Venn diagram of functional annotations of unigenes in nt (NCBI non-redundant protein sequences), nr (NCBI non-redundant protein sequences), kog (Clusters of Orthologous Groups of proteins), go (Gene Ontology) and pfam (Protein family) databases. (B) Heatmap of expression patterns in control and insect feeding samples.

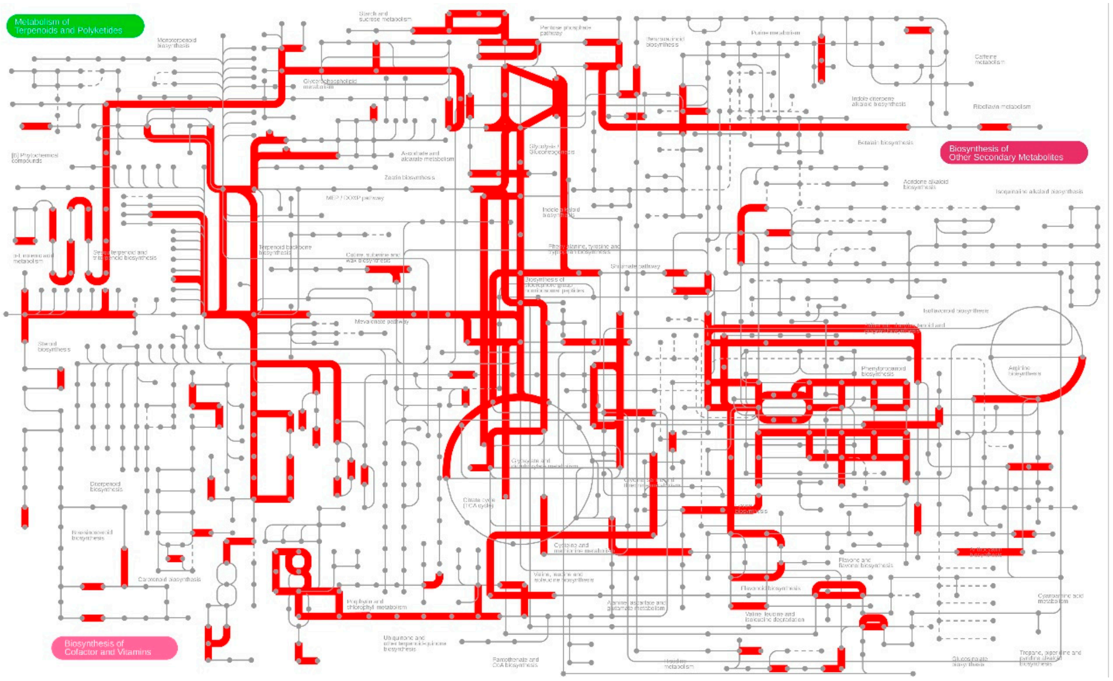

Figure S2. Secondary metabolite pathways enriched by up-regulated genes in mulberry after feeding by the silkworm 3 h. The map was generated with iPath (http://pathways.embl.de), a web-based tool for the visualization of metabolic pathways. The enriched pathways were marked by red lines (P_value < 0.05). The most enriched pathways were "alpha-Linolenic acid metabolism" (21 up-regulated genes), "phenylpropanoid biosynthesis" (25 up-regulated genes), followed by "sesquiterpenoid and triterpenoid biosynthesis" and "mevalonate pathway".

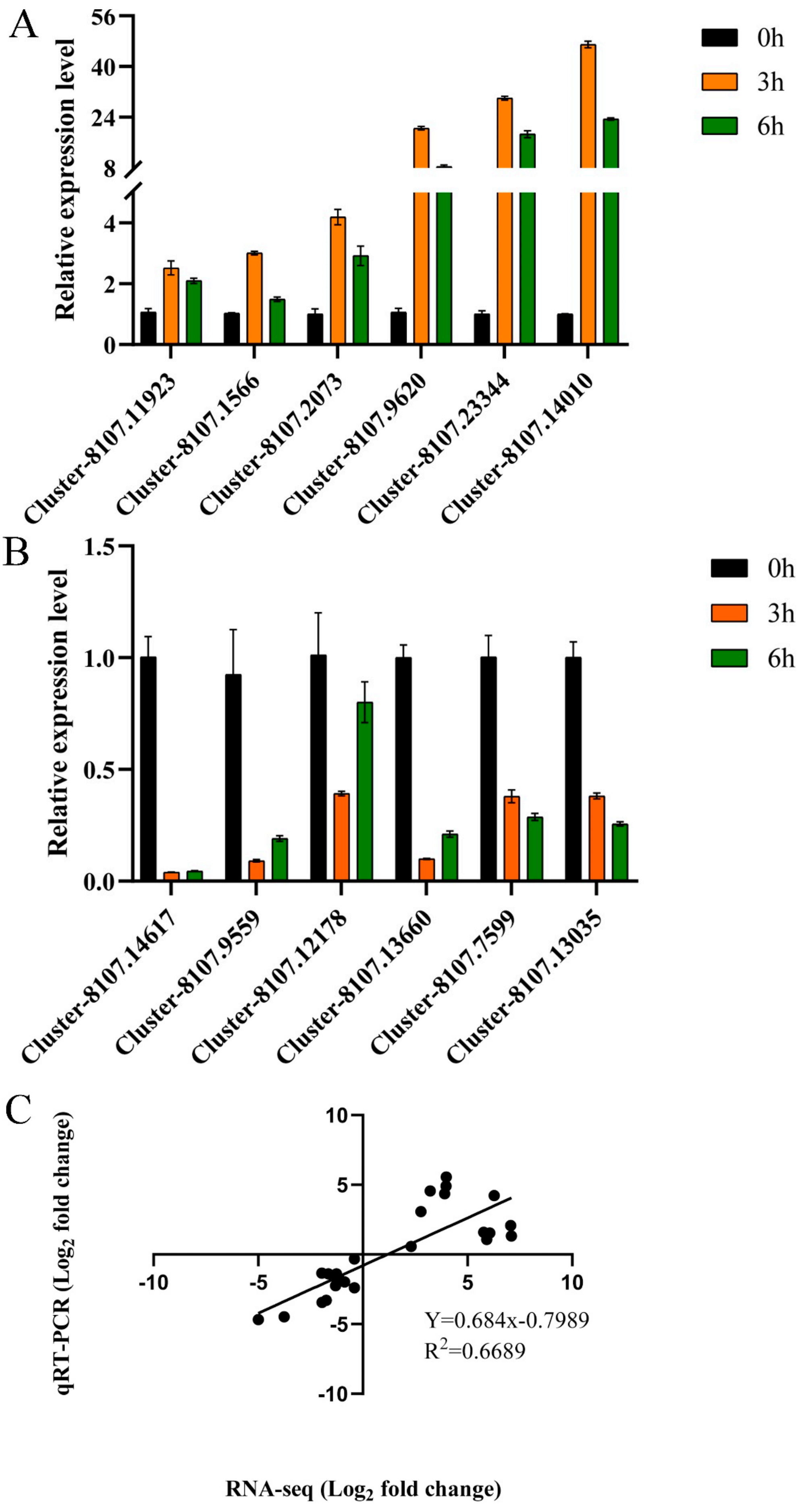
A
Relative expression level
56
40
24
8
4
2
0
0h
3h
6h
Cluster-8107.11923
Cluster-8107.1566
Cluster-8107.2073
Cluster-8107.9620
Cluster-8107.23344
Cluster-8107.14010
B
Relative expression level
1.5
1.0
0.5
0.0
0h
3h
6h
Cluster-8107.14617
Cluster-8107.9559
Cluster-8107.12178
Cluster-8107.13660
Cluster-8107.7599
Cluster-8107.13035
C
qRT-PCR (Log2 fold change)
10
5
-5
-10
-10
-5
5
10
Y=0.684x-0.7989
R2=0.6689
RNA-seq (Log2 fold change)

Figure S3. Validation of RNA-Seq data. Verification of the expression level of the selected eight DEGs from RNA-Seq data through RT-qPCR: (A) Six up-regulated genes (Cluster-8107.11923, Cluster-8107.1566, Cluster-8107.2073, Cluster-8107.9620, Cluster-8107.23344, Cluster-8107.14010). (B) Six down-regulated genes (Cluster-8107.14617, Cluster-8107.9559, Cluster-8107.12178, Cluster-8107.13660, Cluster-8107.7599, Cluster-8107.13035). Error bars indicate the standard error as mean + SD. The x axis represents the relative expression level, and the y-axis represents cultivars. (C) Correlation coefficient of gene expression between qRT-PCR analysis and RNA-Seq data.
